# Fasciolosis, a foodborne zoonotic trematode infection in cattle in Bangladesh: multifaceted validation of parthenogenecity and anthelmintic efficacy

**DOI:** 10.1051/parasite/2026004

**Published:** 2026-02-11

**Authors:** Haydar Ali, Shahadat Hossain, Sharmin Shahid Labony, Aminul Islam, Mohammad Mehedi Hasan, Anita Rani Dey, Mahmudul Alam, Abu Hadi Noor Ali Khan, Abdul Alim, Anisuzzaman

**Affiliations:** 1 Department of Parasitology, Bangladesh Agricultural University Mymensingh 2202 Bangladesh; 2 Department of Pathology and Parasitology, Hajee Mohammad Danesh Science and Technology University Dinajpur 5200 Bangladesh; 3 Department of Medicine, Bangladesh Agricultural University Mymensingh 2202 Bangladesh; 4 Department of Fisheries Technology, Bangladesh Agricultural University Mymensingh 2202 Bangladesh; 5 Department of Surgery and Obstetrics, Bangladesh Agricultural University Mymensingh 2202 Bangladesh; 6 Department of Pathology, Bangladesh Agricultural University Mymensingh 2202 Bangladesh

**Keywords:** *Fasciola gigantica*, Parthenogenic *Fasciola*, Liver fluke, Carboxylesterase B mutation

## Abstract

Parthenogenic *Fasciola* (Trematoda: Fasciolidae) flukes have been developed by the hybridization of *Fasciola hepatica* and *Fasciola gigantica*. They are aspermic (asF) but capable of clonal expansion through parthenogenesis and are spreading rapidly throughout the globe. Here, we unambiguously prove the occurrence of parthenogenic *Fasciola* in cattle in Bangladesh*,* along with their *ex vivo* culture protocol and anthelmintic efficacy. By employing multiple conventional and molecular tools, we confirmed the presence of both the spermic *F. gigantica* (sFg) (31.7%; 814/2575) and asF (68.3%; 1761/2575) in Bangladesh. Both the adult sFg and asF survived well in DMEM supplemented with 20% bovine serum and 20% bovine bile. Using a DMEM-based *ex vivo* culture protocol, we found that nitroxynil (NTX) and oxyclozanide (OCZ) efficiently killed both sFg and asFg in a concentration and time-dependent manner. Surprisingly, triclabendazole (TCBZ) and clorsulon (CRL) killed neither sFg nor asF. Also, praziquantel, albendazole, and levamisole did not affect the viability of the flukes. We found that all TCBZ survivors had more than one mutation, both in nucleotides (G440A, G643A, and G788A) and amino acids (R147K, E215K, and R263K) of the binding pocket of carboxylesterase B (*CestB*), providing molecular evidence of TCBZ resistance in *Fasciola*. Taken together, asF constitutes more than two-thirds of the *Fasciola* population in Bangladesh. This study unambiguously proved the ineffectiveness of TCBZ against both asF and sFg circulating in Bangladesh. Therefore, only OCZ and NTX remain effective against fasciolosis, which thus poses ongoing public health risks of infection in humans with TCBZ-tolerant strains of fasciolosis.

## Abstract (fr)

Les douves *Fasciola* parthénogéniques (Trematoda : Fasciolidae) sont issues de l’hybridation de *Fasciola hepatica* et *Fasciola gigantica* ; elles sont aspermiques (asF), capables de se multiplier par parthénogénèse et se propagent rapidement à travers le monde. Dans cette étude, nous démontrons sans ambiguïté la présence de *Fasciola* parthénogénétique chez les bovins au Bangladesh, et décrivons son protocole de culture *ex vivo* ainsi que l’efficacité des anthelminthiques. Grâce à l’utilisation de plusieurs outils conventionnels et moléculaires, nous avons confirmé la présence de *F. gigantica* spermatique (sFg) (31,7 % ; 814/2575) et asF (68,3 % ; 1761/2575) au Bangladesh. Les douves adultes sFg et asF ont toutes deux bien survécu dans du DMEM supplémenté avec 20 % de sérum bovin et 20 % de bile bovine. Grâce à un protocole de culture *ex vivo* en milieu DMEM, nous avons constaté que le nitroxynil (NTX) et l’oxyclosanide (OCZ) tuaient efficacement les deux types de douves, de manière concentration-dépendante et temps-dépendante. De façon surprenante, le triclabendazole (TCBZ) et le clorsulon (CRL) n’ont eu aucun effet sur les deux espèces. Par ailleurs, le praziquantel, l’albendazole et le lévamisole n’ont pas affecté la viabilité des douves. Nous avons constaté que tous les survivants au TCBZ présentaient plusieurs mutations, tant au niveau des nucléotides (G440A, G643A et G788A) que des acides aminés (R147K, E215K et R263K) du site de liaison de la carboxylestérase B (*CestB*), ce qui constitue une preuve moléculaire de la résistance au TCBZ chez *Fasciola*. La souche asF représente plus des deux tiers de la population de *Fasciola* au Bangladesh. Cette étude a démontré sans ambiguïté l’inefficacité du TCBZ contre les souches asF et sFg circulant au Bangladesh. Par conséquent, seuls l’OCZ et le NTX restent efficaces contre la fasciolose, ce qui représente un risque persistant pour la santé publique lié à l’infection humaine par des souches de fasciolose tolérantes au TCBZ.

## Introduction

Fasciolosis is a highly pathogenic and zoonotic parasitic disease caused by the trematode species, *Fasciola hepatica* and *Fasciola gigantica*, belonging to the family Fasciolidae (Trematoda: Fasciolidae) [23]. These parasites are found in more than 70 countries on all continents, except Antarctica. *Fasciola hepatica* is predominately found in temperate climates, but is also prevalent in tropical and subtropical countries, including in the Middle East (Egypt and Iran), South America (Bolivia, Ecuador, and Peru) and Asia. On the other hand, *F. gigantica* is primarily found in the tropical regions of Asia, Africa, and the Middle East [23]. However, both *F. hepatica* and *F. gigantica* are found in Egypt, Armenia, Niger, Algeria, South Africa, and Iran. A so called intermediate or hybrid population develops due to inter-species cross fertilization between *F. hepatica* and *F. gigantica,* which is aspermic and unable to carry out parthenogenic reproduction and is often reported particularly in the areas where both species co-exist [1, 23, 30]. In contrast, parthenogenic or aspermic *Fasciola* (asF) developed in ancient China, originally due to hybridization of *F. hepatica* and *F. gigantica*. The resulting progeny lost their spermatogenic capability, but became able to follow parthenogenesis and undergo clonal expansion [11]. Currently, the asF is prevalent in different countries such as China, South Korea, Japan, Vietnam, Thailand, Myanmar, Nepal, India, and Bangladesh [11].

Like other trematodes, the lifecycle of *Fasciola* spp. is very complex and require a vertebrate as the definitive host for sexual reproduction and a freshwater snail (Lymnaeidae) as an intermediate host for asexual reproduction. Adult flukes reside in the biliary ducts and gall bladder of the definitive hosts, where they can release up to 25,000 eggs a day. Eggs pass through feces into the environment and hatch to a miracidium, which infects fresh water snails. Inside the snail, the parasite undergoes asexual development and finally a large number of cercariae emerge from the snail. The freely swimming cercariae encyst either on leafy vegetables or at the water surface to form the resistant metacercariae (MC), the infective stage. *Fasciola* spp. infection only occurs when the mammalian hosts, including humans ingest MC with aquatic vegetation or MC-contaminated water [2, 39]. In the small intestine, newly excysted juveniles (NEJs) are released from MC and penetrate the gut wall. Then, NEJs penetrating Glisson’s capsule migrate through the peritoneal cavity and reach the liver by one-week post infection. NEJs move through to the liver parenchyma and grow significantly by feeding on host tissue cells and blood. After 3 to 4 months, the parasites reach the bile ducts where they develop into sexually mature adults and start laying eggs [19]. Liver flukes damage liver parenchyma during migration that severely destroy hepato-biliary tracts and eventually lead to fibrosis. The disease can occur both in acute and chronic forms; however, the chronic form is more common, and manifests as anemia, liver dysfunction, weight loss, fever, nausea, hepatomegaly, skin rash, extreme abdominal pain, and often death [13]. In addition to emaciation, stunted growth and generalized edema, followed by bottle jaw, are the common features of fasciolosis in ruminants. Also, the flukes have been reported to cause anestrus and conception failure in livestock that accounts for substantial economic losses [3, 16, 21, 25, 37].

Up to now, an effective vaccine against fasciolosis is yet to be commercialized; therefore, control of the fluke is mainly dependent on prophylactic chemotherapy by anthelmintics. The commonly used flukicides for treatment of *Fasciola* infection are triclabendazole (TCBZ), nitroxynil (NTX), oxyclozanide (OCZ), and clorsulon (CRL), which have a different spectrum of activity against immature and mature *Fasciola* spp. [28]. Genetic analyses of *Fasciola* isolates throughout the world have shown high levels of genetic heterogeneity, which may play a role in the development of anthelmintic resistance (AR) [7, 14, 15, 21, 22]. To date, the majority of studies have investigated the development of AR in ruminant liver flukes, and studies aim to demonstrate the existence and spread of TCBZ resistance since TCBZ is the most commonly used fasciolicide both in humans and livestock [9, 15]. Although AR against sFg and asF has not been detected in Bangladesh, anthelmintic treatment targeting fasciolosis has recently provided equivocal results that warrant the detection of AR in both sFg and asF. Furthermore, it is essential to develop a standard *ex vivo* culture protocol for *Fasciola* spp., which will be extremely helpful for new drug discovery, screening of immune sera, understanding the developmental biology, and determining the efficacy of commercially available drugs. Consequently, the *ex vivo* culture method will minimize the use of experimental animals, and will ensure Replacement, Reduction, and Refinement (3Rs) of animal use. Importantly, anthelmintic efficacy to asF has not yet been conclusively detected globally. In the present study, we unambiguously confirmed the prevalence of both sFg and asF in Bangladesh along with the efficacy of the commonly used flukicides against both forms of *Fasciola* in Bangladesh. We also developed and optimized an *ex vivo* culture system for *Fasciola* spp.

## Materials and methods

### Ethics considerations

Our experimental protocols were reviewed and approved by the Animal Welfare and Experimentation Ethics Committee of Bangladesh Agricultural University, Mymensingh [Approval number: AWEEC/BAU/2021(37)]. All the experimental procedures were conducted following the guidelines given by the ethics committee.

### Study area and period

**Figure 1:**
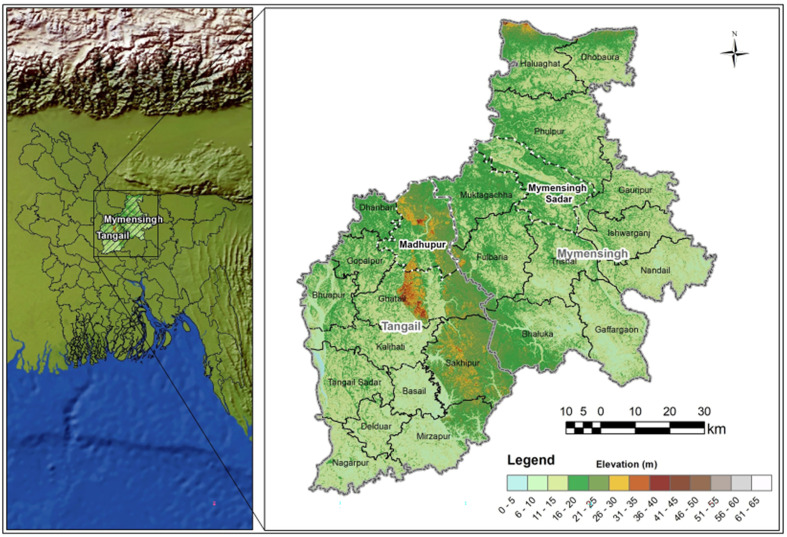
Map showing the study areas.

The study was conducted in two different areas: Mymensingh (Co-ordinates: 24°38′3″N 90°16′4″E, the Bramhaputra alluvium and flood-prone low-lying areas) and Madhupur (Co-ordinates: 24° 37′ 0.12″N 90° 01′ 30.00″E, the red soil tract and highland areas) of Bangladesh from July 2022 to June 2024 (Fig. 1). During selection of animals, the source of animals was confirmed by questioning the owner of the slaughterhouse. Imported cattle, if any, were not included for sampling.

### Sample collection, examination, and parasite identification

We conducted a slaughterhouse based cross-sectional study by randomly selecting live cattle of both sexes, different age groups, and breeds. Immediately after slaughter, livers and gallbladders were examined grossly by close inspection and digital palpation for the presence of lesions produced by liver flukes. A total of 386 animals were examined, and 2,575 flukes were recovered from the infected livers. To isolate flukes, livers were dissected along the course of the bile ducts. Then the liver tissues were cut into small pieces, and kept in normal saline for the release of the flukes remaining in the bile duct. The gallbladder was also opened, inspected, and flukes were collected, if any. The recovered liver flukes were washed in phosphate buffered solution (PBS) and identified by preparing permanent slides, as described previously [39]. The liver flukes were also preserved individually in absolute ethanol for molecular analysis.

### Morphometric detection of different forms of *Fasciola*

Length and width of each adult fluke were measured using a normal scale without stretching them. Data regarding the length and width of flukes were plotted onto an excel sheet and average body length and width were computed. Therefore, the body score (BS; BS = length/width) of each fluke was calculated by dividing body length by width.

### Histological analysis of flukes

Flukes (both sFg and asF) were flattened under gentle pressure and fixed with Carnoy’s solution (ethanol and glacial acetic acid at 3:1 ratio) for 48 h. Then thin sections (5 μm) were made and stained with hematoxylin and eosin (H&E) and examined in a blinded manner.

### Identifications of spermatogonia

Freshly collected flukes (both sFg and asF) were macerated by gentle brushing through a Cell Strainer (pluriStrainer^®^ 300 μm, Thermo Fisher Scientific, Waltham, MA, USA) in normal saline. Then the suspension derived from each fluke was collected in a 50 mL Falcon tube and kept for 10 min. Then the supernatant was centrifuged at 1,000 rpm for 3 min. After centrifugation, supernatant was discarded, and thin smear was prepared with the pellet and stained with Giemsa’s stain. Each slide was examined in a blinded manner.

### Genomic DNA (gDNA) extraction, PCR and visualization

For the validation of *Fasciola* species, small portion from cephalic cone of each fluke was taken and gDNA of the fluke was extracted using QIAmp Mini Kit (QIAGEN, Hilden, Germany), following the manufacturer’s instructions. Then PCR was conducted by employing specific primers (Supplementary Table 1) targeting the internal transcribed spacer 1 (*ITS1*) gene*.* The PCR reaction was performed in a total volume of 25 μL with 12.5 μL of master mix (OneTaq^®^ Quick Load, New England Biolabs, Inc., Ipswich, MA, USA) containing polymerase enzyme, dNTP and MgCl_2_, and 1 μL of 10 pmol of each primer and 100 ng of gDNA. PCR cycles for *ITS1* consisted of an initial denaturation at 94 °C for 90 s, followed by 30 cycles at 94 °C for 90 s, 55 °C for 90 s, and 72 °C for 120 s, with a final extension at 72 °C for 10 min [12]. Then the PCR products were electrophoresed in 1.5% agarose gels (MP Biochemicals LLC, Illkirch, France), and DNA bands were visualized in UV-light using ethidium bromide.

### Phosphoenolpyruvate carboxykinase (*pepck*)-based multiplex PCR

To validate the morphometry-based identification of sFg and asF, we conducted previously established *pepck*-based multiplex PCR with specific primers (Supplementary Table 1). The PCR was conducted in a total volume of 25 μL which contained 12.5 μL of master mix (OneTaq^®^ Quick Load), 1 μL of 10 pmol of each forward primer (Fh-pepck-F and Fg-pepck-F), 2 μL of 10 pmol of common reverse primer (Fcmn-pepck-R) and 100 ng of gDNA. PCR cycles consisted of an initial denaturation at 94 °C for 1.5 min, followed by 30 cycles at 94 °C for 30 s, 61 °C for 30 s, and 72 °C for 1 min, with a final extension at 72 °C for 10 min [40]. PCR products were electrophoresed and visualized following the same procedures as mentioned above.

### *Ex vivo* culture of sFg and asF

The collected asF (*n* = 74) and sFg (*n* = 74) were washed in sterile PBS supplemented with 200 U/mL penicillin, and 200 mg/mL streptomycin (Sigma-Aldrich, Darmstadt, Germany). Adult flukes (2 parasites in 2 mL) were incubated in Medium 199 (M199, (Sigma-Aldrich), DMEM (Sigma-Aldrich) or in RPMI 1640 (Sigma-Aldrich) supplemented with bovine serum (BS, Cytiva, HyClone Laboratories, Logan, UT, USA), bovine bile (BB, collected from specific pathogen-free cattle) at different concentrations (5–20%) along with 200 U/mL penicillin, and 200 mg/mL streptomycin (Sigma-Aldrich) in a 12-well flat bottom cell culture plate (Corning Incorporated, Corning, NY, USA) and incubated at 37 °C in 5% CO_2_ in a humidified air up to 48 h. For each condition, experiments were performed in triplicate. The viability parameters of flukes were assessed under an inverted microscope (Labomed Inc., Los Angeles, CA, USA) at 0, 3, 6, 12, 24, 36, and 48 h of incubation. The culture medium was changed at every 12 h interval. Viability was evaluated in a blinded manner observing motility, pharyngeal pump, flow of intestinal contents and integrity of the cuticle (Supplementary Table 2). Flukes kept only in the respective culture media served as a control.

### *Ex vivo* egg harvesting and morphometry

Adult flukes (sFg, *n* = 10; asF, *n* = 10) were maintained overnight in DMEM supplemented with 20% of BS and 20% of BB, and incubated overnight in the same condition as mentioned above. The following morning, eggs laid by sFg and asF were collected separately by pipetting and examined in a blinded manner. The length and width of 100 eggs from each group were estimated.

### *Ex vivo* detection of anthelmintic efficacy

Technical grade anthelmintics such as NTX, OCZ, TCBZ, praziquantel (PZQ), CRL, levamisole (LEV), and albendazole (ABZ) were dissolved in dimethyl sulfoxide (DMSO, Wako Pure Chemical Industries LTD, Osaka, Japan) or PBS, and stored at −20 °C. The asF or sFg were maintained 1 h in DMEM supplemented with 20% BS and 20% BB in the same condition as mentioned above. After 1 h, the selected anthelmintics were added to the culture medium at different concentrations (2.5–40 μg/mL for NTX (Sk+F, Dhaka, Bangladesh) and OCZ (Sk+F, Dhaka, Bangladesh); 20–200 μg/mL for TCBZ (Rakshit Drugs Private Limited, Hyderabad, India), PZQ (Merck, Rahway, NJ, USA), albendazole (ABZ; Pharmacon Vet PVT. Ltd., Kolkata, India) and CRL (Ballygunge Chemical, Kolkata, India); 10–20 μg/mL for LEV (Pharmacon Vet PVT. Ltd., Kolkata, India), and were incubated further up to 36 h. For each condition, experiments were performed in triplicate. Untreated adult sFg or asF kept in culture media containing DMSO (10 μL/mL) served as controls. Scoring was performed at 0, 3, 6, 12, 24, and 36 h post treatment (p.t) in a blinded manner.

### Carboxylesterase B (CestB)-based touchdown PCR (TD-PPCR) for the molecular validation of TCBZ resistance

Very recently, single nucleotide polymorphisms (SNPs) in the *Cestb* gene has been found to be linked to TCBZ resistance [39, 41]. Therefore, we isolated gDNA from the cephalic cone region of *ex vivo* TCBZ-tolerant flukes (both sFg and asF), following the procedures mentioned above. Then, TD-PCR was conducted using the following primers (forward: FExon1CestB 5′–CGGGTCCAAGCAAGGATGAG–3′; reverse: RExon1CestB 5′–CTCTCCTCCGACCATCAAATTC–3′) in a total volume of 25 μL containing 12.5 μL of master mix (OneTaq^®^ Quick Load, USA), 1 μL of 10 pmol of each primer and 100 ng of gDNA. The thermal cycle consisted of 3 min of denaturation at 95 °C, followed by 10 cycles at 94 °C for 15 s, 65 °C for 30 s, and 72 °C for 30 s, and programmed to subtract 1 °C from each cycle to the annealing step followed by 15 cycles of 93 °C denaturation for 30 s, 60 °C annealing for 30 s and 72 °C extension for 40 s and a final extension step of 72 °C for 5 min. PCR products were visualized by ethidium bromide.

### Sequencing and bioinformatics analysis

PCR products derived from *CestB* gene-based analysis were randomly selected (sFg, *n* = 6 and asF, *n* = 4) and column purified. Purified products were sequenced commercially in both forward and reverse directions. Sequences were aligned and edited using the BioEdit 7.2 software. The obtained sequences have been submitted to the GenBank (accession numbers: PQ231166–PQ231175). Sequences were compared against the NCBI database using BLASTN (https://blast.ncbi.nlm.nih.gov/Blast.cgi). The obtained DNA sequences were translated to the respective amino acid sequences by ORFinder at NCBI (https://www.ncbi.nlm.nih.gov/orffinder/). The Clustal Omega online algorithm was used for multiple sequence alignment of both DNA and amino acid sequences obtained, and for localization of SNPs and amino acid substitution (https://www.ebi.ac.uk/Tools/msa/clustalo/).

## Statistical analysis

All collected data were encoded into a Microsoft Excel spreadsheet. The Z-test was performed to determine the influence of study area. The one-way ANOVA followed by Bonferroni *post hoc* analysis was conducted to analyze the data regarding anthelmintic efficacy and a *p* < 0.05 was considered significant. Mixed infections were analyzed by preparing a Venn Diagram.

## Results

### Presence of parthenogenic fasciolid flukes in Bangladesh

**Figure 2:**
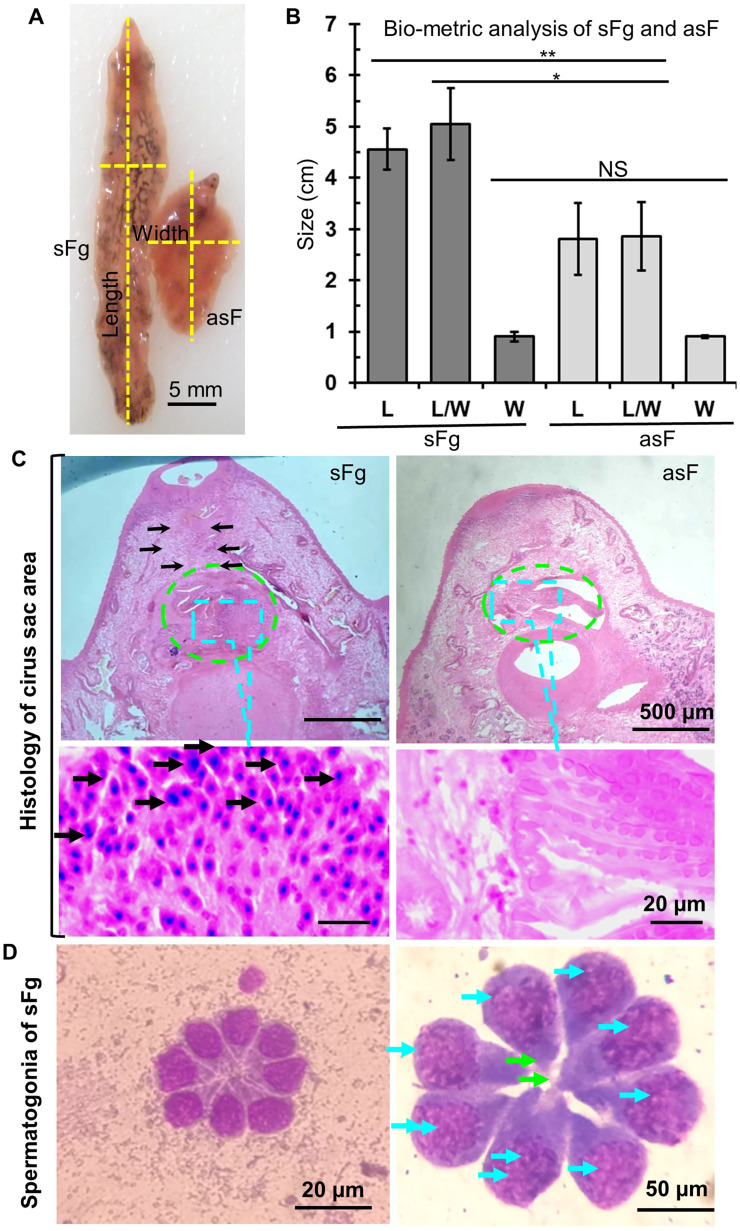
Morphological and morphometric identification of spermic *Fasciola gigantica* and aspermic (parthenogenic) *Fasciola*. (A) Aspermic/parthenogenic *Fasciola* and spermic *Fasciola gigantica*. sFg, spermic; asF, aspermic (parthenogenic) *Fasciola*. (B) Biometric analysis of sFg and asF. L, length; W, width; NS, non-significant. (C) Histology of cirrus sac. Dotted green circle is the cirrus sac. In the bottom panels, the yellow arrow indicates nuclei of the sperm. (D) Testicular tissues isolated from the sFg. In the right panel, the yellow arrows indicate spermatogonia.

**Supplementary Figure 1:**
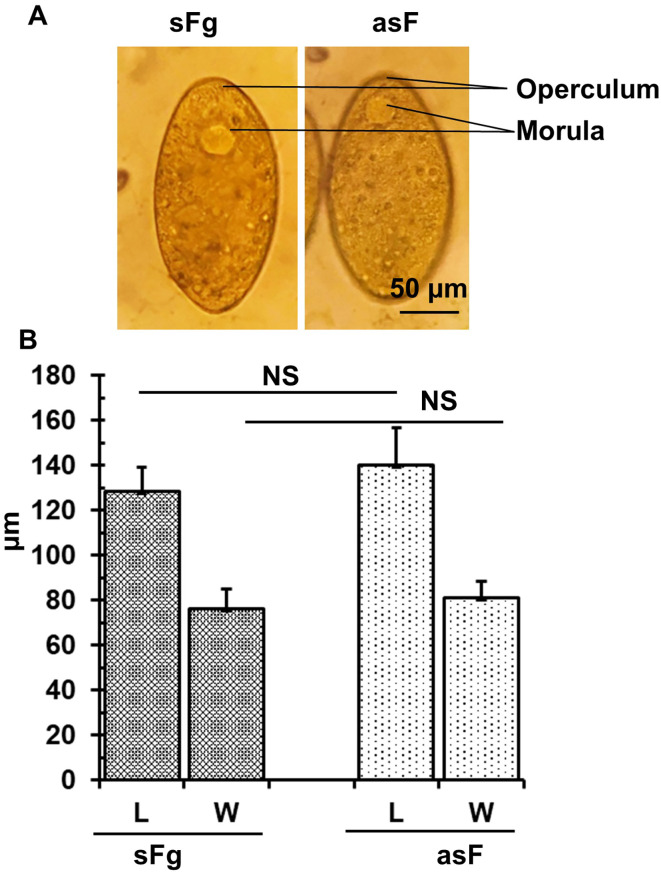
Morphology and morphometry of eggs laid by aspermic *Fasciola* (asF) and spermic *Fasciola gigantica* (sFg). Adult flukes were incubated in DMEM supplemented with 20% bovine serum, 20% bovine bile along with 200 U/mL penicillin, and 200 mg/mL streptomycin in a 12-well flat bottom cell culture plate. Eggs were collected the following morning. (A) Microphotograph of the eggs laid by asF and sFg. (B) Size of eggs laid by asF and sFg*.*

**Supplementary Figure 2:**
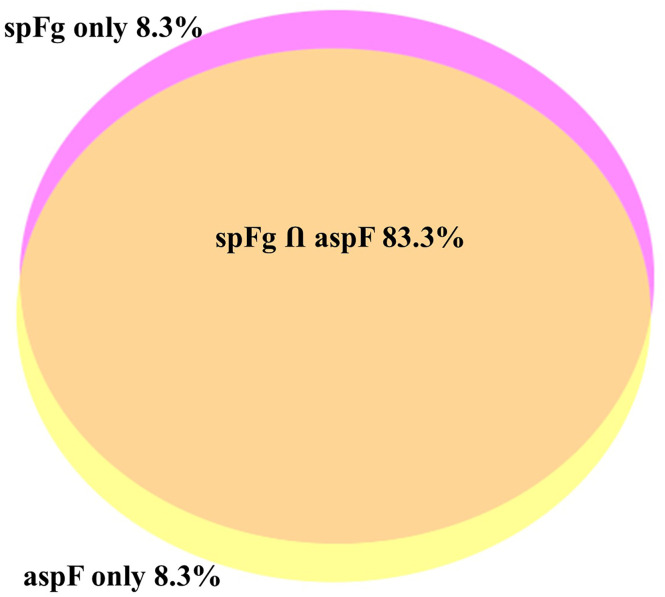
Venn diagram showing mixed infection with aspermic *Fasciola* (asF) and spermic *Fasciola gigantica* (sFg).

By detailed morphometric analysis, we found that both sFg and asF were prevalent in Bangladesh. During our study, we isolated and analyzed 2,575 adult liver flukes and the number of flukes isolated from each animal varied widely, ranging from 2 to 328 flukes per liver. On the basis of morphometric analysis, we found a significantly (*p <* 0.05) higher number of asF (68.3%, 1761/2575) than sFg (31.7%, 814/2575). Average size of the sFg was 4.3 ± 0.4 × 0.9 ± 0.1 cm, whereas asF was 2.3 ± 0.5 × 1.1 ± 0.1 cm (Fig. 2A). However, like sFg, the uterus of the asF contained fully developed eggs, suggesting that the relatively smaller asF were not immature flukes. At present, asF were easily distinguished by calculating BS (BS = BL/BW), even without microscopic examination of the presence or absence of sperm in the cirrus sac. *Fasciola* spp. with BS 1.6, 2.5–3.5 and >4.0 are considered to be spermic *Fasciola hepatica* (sFh), asF, and sFg, respectively [11]. We estimated BS of each of the recovered flukes (2,575) and we found two distinct categories of flukes: (I) flukes with high BS ranging from 4.5 to 7.0 (5.1 ± 0.7) and (II) flukes with low BS ranging from 1.9 to 3.5 (2.9 ± 0.7) (Fig. 2B), suggesting further that both sFg and asF are prevalent in Bangladesh. Histologically, sFg was characterized by the presence of sperm in the cirrus sac, but asF did not have any sperm (Fig. 2C). Isolation and examination of testicular tissues from both asF and sFg revealed the presence of spermatogonia in sFg only (Fig. 2D). On the other hand, we did not find any spermatogonia in asF, suggesting absolute loss of production of sperm in asF. However, we could not detect any significant (*p* > 0.05) differences in the size and shape of the eggs derived from the culture of asF and sFg (Supplementary Figs. 1A, 1B), which indicated that identification of asF and sFg was not possible by the microscopic examination of feces collected from infected animals. Venn diagram revealed that most of the examined cattle (83.3%) were infected with both asF and sFg (Supplementary Fig. 2).

### Molecular validation of asF in Bangladesh

**Figure 3:**
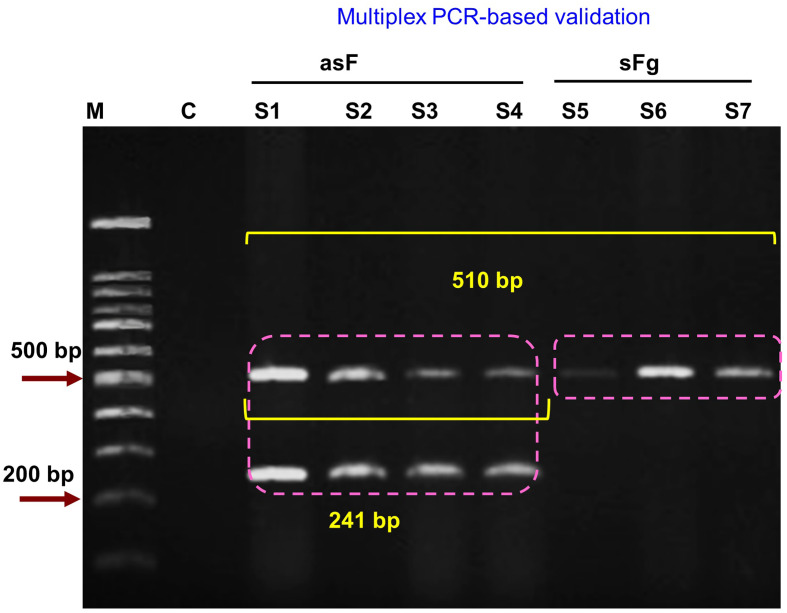
Multiplex PCR-based confirmation of aspermic/parthenogenic *Fasciola* and spermic *Fasciola gigantica*. A *pepck*-based multiplex PCR was conducted and PCR products were electrophoresed. Double bands were detected asF, aspermic (parthenogenic) *Fasciola* and a single was detected in sFg, spermic *Fasciola gigantica.*

Initially, we conducted the commonly used *ITS1*-based PCR, which was successful, confirming that the flukes belonged to the genus *Fasciola*. *ITS1*-based PCR amplified both sFg and asF, but produced amplicons at the same level (680 bp); therefore, it failed to differentiate the types of the fasciolid flukes. Then using the well-established *pepck*-based multiplex PCR, we unambiguously demonstrated that both asF and sFg, but not *F*. *hepatica*, were prevalent in Bangladesh. We obtained a single band at the ~510 bp level from the PCR that was conducted using gDNA isolated from sFg, but PCR revealed two bands (at ~510 bp and ~240 bp), while the gDNA isolated from asF was used (Fig. 3), confirming our morphometric and histology-based identification of sFg and asF, respectively.

### Selection of conditions for *ex vivo* culture of liver flukes

**Figure 4:**
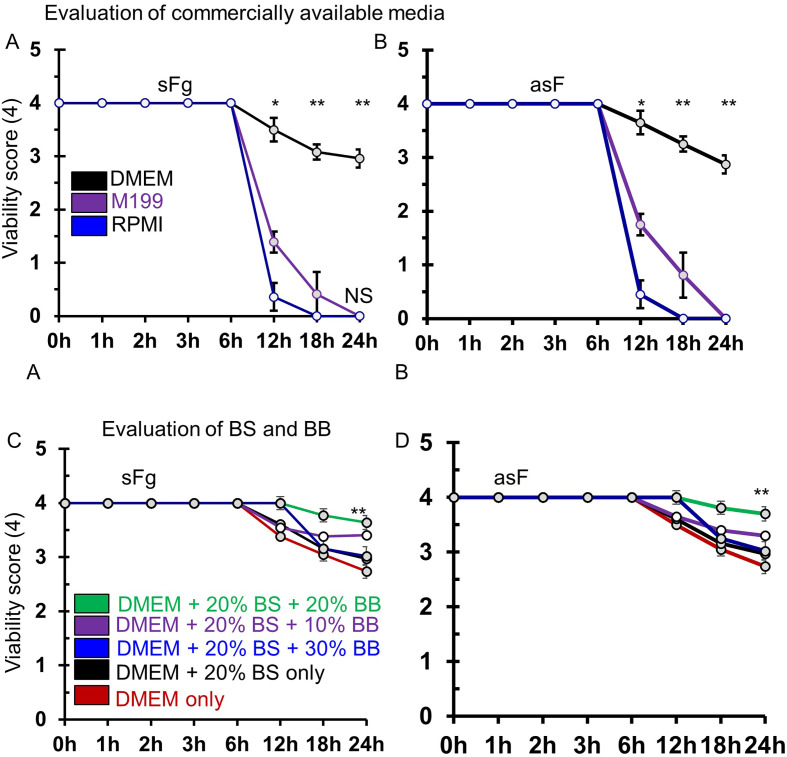
Evaluation of *ex vivo* culture condition for both spermic *Fasciola gigantica* (sFg) and aspermic (parthenogenic) *Fasciola* (asF)*.* (A) Survival of the sFg in different commercially available media. Adult sFg (2 parasites in 2 mL) were incubated in different media (M199, DMEM or RPMI 1640) containing 200 U/mL penicillin, and 200 mg/mL streptomycin in a 12-well flat bottom cell culture plate. Parasites were scored in a blinded manner, as mentioned in the Materials and Methods section. (B) Survival of asF in the commercially available media. Adult asF (2 parasites in 2 mL) were incubated following the same procedures as mentioned above and scored. (C) Adult sFg (2 parasites in 2 mL) were incubated in DMEM supplemented with bovine serum (BS), bovine bile (BB) at different concentrations (5–20%) along with 200 U/mL penicillin, and 200 mg/mL streptomycin in a 12-well flat bottom cell culture plate in the same condition, and were scored. (D) Adult asF (2 parasites in 2 mL) were incubated in DMEM supplemented with BS, BB at different concentrations following the identical procedures as mentioned above and scored. Each experiment was conducted in triplicate and data are presented as mean ± SE. *p < 0.05; **p < 0.01.

On the basis of collective scoring, the present study revealed that RPMI medium failed to provide sufficient support for the survival of the both sFg and asF more than 6 h. In this medium, viability of both types of flukes dropped rapidly at 6 h and declined to 0.5 (out of 4) at 12 h. In the RPMI medium, all parasites (both sFg and asF) died by 18 h. Also, M199 media failed to provide sufficient support for fluke survival, whether sFg or asF, and all parasites died by 18 h. On the other hand, DMEM efficiently supported the survival and reproduction of sFg and asF, and the viability score remained >3.5 at even 12 h of incubation, then gradually declined to >3 by 24 h (Figs. 4A, 4B). As a result, we decided to use DMEM for the establishment of the *ex vivo* culture protocol. In the next experiment, we found that addition of BS (20%) to DMEM significantly (*p <* 0.05) increased the viability of sFg and asF, and they survived up to 12 h with a viability score of 4.00, which declined to <3 by 18 h. Notably, addition of BB (20%) in the BS (20%) supplemented DMEM media, also significantly (*p <* 0.05) increased the viability of the flukes. In this combination, both sFg and asF survived up to 12 h with the viability score of 4.00, and up to 24 h with the viability score of 3.64 (out of 4) (Figs. 4C, 4D). However, further increase of bile did not improve fluke *ex vivo* viability (data not shown).

### Efficacy of anthelmintics against both sFg and asF

**Figure 5:**
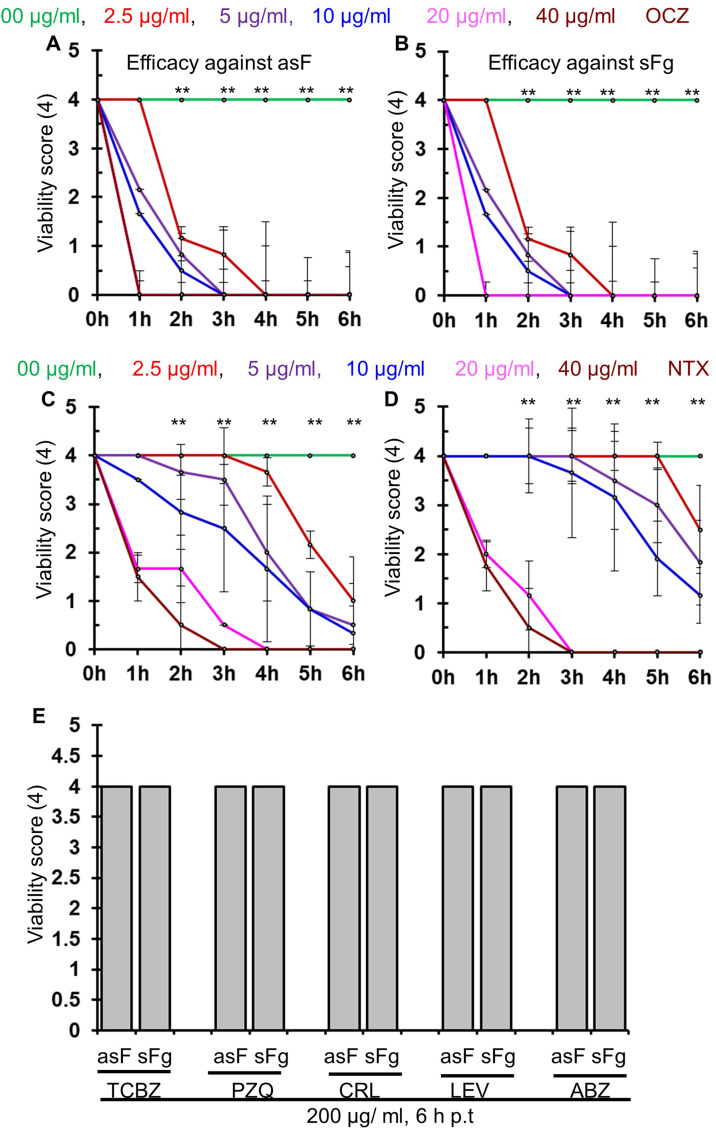
Efficacy of commercially available anthelmintics against both spermic *Fasciola gigantica* (sFg) and aspermic/parthenogenic *Fasciola* (asF). The flukes were maintained 1 h in DMEM supplemented with 20% of bovine serum (BS) and 20% of bovine bile (BB) by adding with 200 U/mL penicillin, and 200 mg/mL streptomycin, following the same procedure as mentioned above. Then the selected anthelmintics were added to the culture medium at different concentrations and were incubated further. Untreated *Fasciola* spp. kept in media containing DMSO served as the control. Scoring was performed at 0, 3, 6, 12, 24, and 36 h post treatment (p.t) in a blinded manner. (A) Concentration dependent efficacy of oxyclozanide against asF*.* (B) Concentration dependent efficacy of oxyclozanide against sFg*.* (C) Concentration dependent efficacy of nitroxynil against asF*.* (D) Concentration dependent efficacy of Nitroxynil against sFg*.* (E) Efficacy of triclabendazole and other anthelmintic against both sFg and asF. Each experiment was conducted in triplicates and data were presented as mean ± SE. TCBZ, triclabendazole; OCZ, Oxyclozanide; NTX, Nitroxynil; PZQ, Praziquantel; CRL, Clorsulon; LEV, Levamisole, ABZ, Albendazole*.* *p < 0.05; **p < 0.01.

Using DMEM supplemented with 20% BS plus 20% BB in the *ex vivo* culture protocol, we tested the efficacy of the commonly used and commercially available anthelmintics. During our study, we found that NTX and OCZ efficiently killed both sFg and asF, in a concentration and time dependent manner. At 40 μg/mL concentration, NTX killed both types of flukes within 3 h (Figs. 5A, 5B); however, OCZ killed the flukes even more efficiently within 1 h (Figs. 5C, 5D). Surprisingly, TCBZ and CRL did not affect the viability score of the treated flukes. Even at 200 μg/mL concentration of TCBZ and CRL, both sFg and asF survived with a viability score of 4 (out of 4) at 6 h p.t. Similarly, PZQ, ABZ, and LEV did not affect the viability of any of the fluke types at the highest concentration used (200 μg/mL) (Fig. 5E).

### SNPs detection at the TCBZ binding pocket of *CestB*

To detect SNPs in the *CestB* gene, we conducted PCR using the previously established protocol and we obtained amplicons at the expected levels (~943 bp). Then PCR products obtained from each fluke was subjected to sequencing. The newly retrieved nucleotide sequences of the *CestB* gene were aligned with the reference sequences of *CestB* sequences of *F. hepatica* (accession number: MW655750.1), which demonstrated SNPs at positions 440, 643, and 788, where “G” was replaced by “A”. Subsequently, we translated the nucleotide sequences into the amino acid sequences. To find out the SAAPs, we aligned the amino acid sequences with the reference amino acid sequences of *CestB* protein. We found SAAPs in the amino acid sequences at positions 147 (R147K), 215 (E215K), and 263 (R2663K) (Table 1).

**Table 1:** List of nucleotide and amino acid polymorphisms in the *CestB* gene of *ex vivo* TCBZ-resistant sFg and asF compared to the reference gene of *CestB* of TCBZ-susceptible *F. hepatica*.

Accession No	Fluke types	*Ex vivo* TCBZ response (Sen/Res)	SNP440	SNP643	SNP788	SAAP147	SAAP215	SAAP263
PQ231166	sFg	Res	A	A	A	K	K	K
PQ231167		Res	A	A	A	K	K	K
PQ231168		Res	A	A	A	K	K	K
PQ231169		Res	A	A	A	K	K	K
PQ231170		Res	A	A	A	K	K	K
PQ231171		Res	A	A	A	K	K	K
PQ231172	asF	Res	A	A	A	K	K	K
PQ231173		Res	A	A	A	K	K	K
PQ231174		Res	A	A	A	K	K	K
PQ231175		Res	A	A	A	K	K	K
MW655750.1 (Reference Gene)		Sen	G	G	G	R	E	R

Sen, sensitive; Res, resistance; TCBZ, triclabendazole; sFg, spermic *Fasciola gigantica*; asF, aspermic *Fasciola*.

## Discussion

Fasciolosis is a food-borne zoonotic disease and has been listed as a neglected tropical disease by the World Health Organization (WHO). The WHO has proposed a “Road Map 2030” to prevent, control, eliminate, or eradicate 20 diseases or disease groups to achieve targets aligned with the Sustainable Development Goals. Until now, control of fasciolosis has solely relied on anthelmintic treatment and, to some extent, on ecological manipulation targeting vector snail control. Unfortunately, only very few anthelmintics are commercially available against fasciolosis and their efficacy is ambiguous. Furthermore, resistance to anthelmintics has been reported from many countries [7, 34, 41]. In addition, the evolution and rapid spread of new variants, such as asF, has further aggravated the situation globally. Here, we report molecular confirmations of asF isolated from naturally infected cattle and we tested the efficacy of commercially available anthelmintics against both sFg and asF.

We found that even in the 21st century, the prevalence of fasciolosis is very high, particularly in the hyperendemic, flood prone, low lying and marshy areas of Bangladesh. In the present study, and for the first time, we have unambiguously shown the prevalence of asF along with sFg, but not *F. hepatica*, in the study areas of Bangladesh by employing multiple conventional methods coupled with the *pepck*-based multiplex PCR. So far, only two species of the genus of *Fasciola* have received global acceptance, *i.e.*, *F. hepatica* and *F. gigantica*. However, a hybrid form of *Fasciola* has been reported in the territories where both the species co-exist, which has developed due to accidental cross-fertilization between the species. The hybrids have neither sperm nor parthenogenetic capability and are therefore unable to produce successive generations. Surprisingly, another form of *Fasciola* known as parthenogenetic or asF is rapidly spreading throughout the globe. “Hybrid form” is completely different from the asF, and by microsatellite DNA analyses, it has been shown that asF are capable for clonal expansion, without self- or cross-fertilization [11]. AsF has been detected in many Asian countries, such as Japan, South Korea, China, Vietnam, Thailand, the Philippines, Myanmar, Nepal, and India [8, 38]. Additionally, *F. hepatica* is prevalent in the countries where its essential molluscan intermediate host, *Galba truncatula* (= *Lymnaea truncatula)* is prevalent, but *F. gigantica* can utilize a range of species of lymnaeid snails such as *Lymnaea auricularia*, *L. luteola*, and *L. viridis*, *etc.* However, asF can utilize intermediate hosts of both *F. hepatica* and *F. gigantica* [11]. In Bangladesh, so far, only *L. auricularia* and *L. luteola*, but not *L. truncatula*, have been detected [17, 18].

We developed a BS and BB supplemented DMEM-based *ex vivo* culture platform where the flukes survived up to 24 h with a high viability score. The main constraint of testing efficacy of new or existing anthelmintics is the lack of a suitable *ex vivo* culture protocol for trematodes, including *Fasciola* spp. Unlike prokaryotic pathogens and even protozoa, trematodes do not grow exponentially in the *ex vivo* culture platform. Similarly, even roundworms (nematodes) can be easily maintained for a long time in *ex vivo* culture media [10, 32], but most of the flukes neither survive for a long time nor develop in the culture media. The present *ex vivo* culture protocol mimics the natural condition to some extent that prevails in the host body. This cell-free culture protocol gave us an opportunity to test the efficacy of anthelmintics without using infected animals. Till today, sensitivity testing of anthelmintics against *Fasciola* spp. is mainly conducted *in vivo*, using naturally or experimentally infected susceptible animals (*e.g.*, sheep, goats, cattle, and buffaloes) through fecal egg count reduction tests [14, 36]. Our protocol will reduce the usage of animals.

Using this *ex vivo* culture protocol, we found that NTX and OCZ efficiently killed both sFg and asF within a very short time. NTX is a nitrate derivative anthelmintic belonging to the group of substituted phenols. It uncouples oxidative phosphorylation in flukes and thereby hampers ATP production, resulting in cellular fuel insufficiency and death [31]. In our study, we found that NTX killed liver flukes by 3 h p.t, which is consistent with the findings of Fairweather *et al.* [34], who observed that NTX caused rapid spastic paralysis of *F. hepatica* by 3 h [6]. NTX can safely be used at any stage of pregnancy and has been reported to eliminate adult liver flukes very efficiently [31]. However, NTX has only been approved for use in veterinary medicine in ruminants (*e.g.*, sheep, goats, cattle, and buffaloes), but is restricted to use in lactating animals. It causes yellow discoloration of milk and its therapeutic index is only four, with a high withdrawal period [42]. On the other hand, OCZ belongs to the salicylanilide group and similarly has been approved only for the treatment and control of fasciolosis in ruminants [43], and is not indicated for humans. The mode of action of the drug is very complicated. Along with uncoupling of oxidative phosphorylation, OCZ rapidly reduces the tegumental pH of the flukes (by 10 min), increases calcium influx in muscle cells, and hampers neuromuscular function, leading to paralysis and starvation by cessation of food intake [33]. During our *ex vivo* trial, we also found that OCZ very rapidly (within 1 h) killed both sFg and asF. In mammalian hosts, OCZ is excreted into the bile and is effective against only adult flukes, but is not active against liver tissue-dwelling immature flukes. However, OCZ had been proved to be well-tolerated in pregnant animals, and no significant untoward effects were detected except loose motion and transient drop of milk production [35].

Most surprisingly, we observed that TCBZ, the main drug of choice against fasciolosis, did not work at all and killed neither sFg nor asF, even within 6 h p.t at higher concentration. TCBZ is a halogenated derivative of benzimidazole (BZD) with excellent efficacy against flukes, particularly against *F. hepatica* and *F. gigantica*. Notably, TCBZ acts against immature flukes and is even effective against 2-day-old early immature flukes [33]. TCBZ is the only BZD that does not kill nematodes; rather, it is highly effective against some trematodes, particularly *Fasciola* spp. It has been reported that β-tubulin mutation at 200 or 167 (phenylalanine to tyrosine) has no impact on the development TCBZ resistant *Fasciola* spp. Recently, it has been shown that CestB protein is associated with the binding of TCBZ, and mutation in the drug binding pocket of the protein, particularly at R147, E215, and R263 confers resistance of *Fasciola* spp. against TCBZ [26]. To reinforce our findings of *ex vivo* culture-based observed TCBZ resistance, we carried out PCR, sequencing of PCR products and subsequent bioinformatic analysis, which unveiled SNPs and SAAPs at the aforementioned positions, suggesting that further TCBZ-resistance in *Fasciola* spp. may be present in Bangladesh. In fact, *CestB* is a xenobiotic metabolizing enzyme (XME), which is ubiquitously present in metazoan animals. XMEs play key roles to protect multicellular animals from the toxic effects of harmful chemicals, including the lethal effects of anthelmintics exerted on helminths. In helminths, several XMEs such as cytochrome P450, monooxygenases, dehydrogenases, and *CestB* have been identified. Of the XMEs identified from helminths and other parasites, *CestB* has a spacious and adaptable binding pocket, consisting of R147, E215, and R263. The pocket can interact with diverse classes of molecules, including carbolic esters, phosphate esters, amides, and thioesters [24]. *CestB* is capable of binding to anthelmintics and pesticides of different groups. Very recently, it has been shown that *CestB* can bind with TCBZ [27] and normally can detoxify the drug without hampering its activity. However, conformational changes of the binding pocket of the enzyme are greatly altered due to SAAPs that dramatically increases the affinity of the enzymes to xenobiotics, including TCBZ. It is well established that SAAPs of *CestB* at R147K, E215K, and R263K significantly increase the affinity of *CestB* to TCBZ, resulting in tight binding and sequestration of TCBZ. Therefore, TCBZ becomes chelated, rendering the anthelmintic ineffective or very poorly effective [39]. In addition to the *CestB* gene, recently Beesley *et al.* [4] showed that several proteins, such as membrane transport (*e.g.* ATP-binding cassette family B, ABCB1), transmembrane signaling and signal transduction (*e.g.* GTP-Ras-adenylyl cyclase and EGF-like protein), DNA/RNA binding and transcriptional regulation (*e.g.* SANT/Myb-like DNA-binding domain protein), and drug storage and sequestration (*e.g.*, fatty acid binding protein, FABP) proteins, are the prime candidates for conferring TCBZ resistance. Furthermore, Choi *et al.* [5] identified genomic regions of high differentiation (FST outliers above the 99.9th percentile) that encode genes involved in the EGFR-PI3K-mTOR-S6K pathway and microtubule function. Transcript expression differences were observed in microtubule-related genes between TCBZ-susceptible (TCBZ-S) and -resistant (-R) flukes, which can differentiate between TCBZ-S and -R parasites with ≥75% accuracy.

In Bangladesh, TCBZ is the most widely used anthelmintic, particularly in the livestock industry. In this country, anthelmintics are used mostly by field veterinary assistants and artificial insemination (AI) technicians, without fecal sample examinations. Indiscriminate use of anthelmintics is usually associated with the development of AR [7].

On the other hand, CRL and ABZ are also used against fasciolosis, but in our study, we could not detect any efficacy. Resistance to CRL and ABZ against *Fasciola* spp. has already been reported in different countries [20, 41]. Although PZQ is a good drug against all trematodes, particularly against schistosomes, it is refractory to *Fasciola* spp. [29]. In our settings, we also did not find efficacy of PZQ against sFg or asF. LEV, although indicated for nematodes, is marketed along with other flukicides (*e.g.*, OCZ and TCBZ); therefore, we tested the efficacy of the drugs against both sFg and asF, but the drug alone did not affect fluke viability at all. Unlike anti-microbial resistance (AMR) to bacteria, AR is hereditary. Therefore, resistance in asF is thought to be more detrimental. If it develops, there is no way to alter AR in asF. It is believed that AR can only be altered in unisexual helminths, such as nematodes and schistosomes, or in bisexual trematodes, which are adapted to cross-fertilization. Cross-fertilization provides an avenue to maintain a “refugium”, a population susceptible to anthelmintics. And, a susceptible refugium population of a particular species of helminth can be maintained in a particular area by the alteration of anthelmintics, reducing frequency of deworming and by avoiding mass drug administrations. If a susceptible refugium can be built up then cross-fertilization between “susceptible” and “resistant” populations will produce a heterozygous generation that will be susceptible, because only the homozygous populations are resistant [7]. Most importantly, once asF become resistant, then reversion or loss of resistance is never possible. This is because the subsequent generation derived from the resistant parents through parthenogenesis will be the carbon copy of the parents, meaning that the subsequent generation will be resistant. Anthelmintics become resistant due to several reasons, in particularly due to (i) indiscriminate use of anthelmintics, (ii) use of the same drug for long time, (iii) improper dosing, and (iv) use of low quality drugs [7]. In Bangladesh, there is indiscriminate, unnecessary, improper (without coprological examination and without measuring body weight), and even unlawful use of anthelmintics, especially by non-veterinary field workers, which may play a key role in the development of AR. Alarmingly, if humans become infected with fasciolosis in Bangladesh, the infection will be difficult to treat since TCBZ is the only drug that is recommended against human fasciolosis.

In conclusion, both sFg and asF are prevalent in Bangladesh. The population is at risk due to liver fluke infection causing fatal liver cirrhosis. All anthelmintics commonly used and so far available in Bangladesh are resistant to both sFg and asF, except for NTX and OCZ. Using multi-faceted approaches, we provide solid evidence of the development of TCBZ resistance both in sFg and asFg. In fact, we did not find significant differences in efficacy of anthelmintics against sFg or asF. Our results will be helpful for the treatment of clinical cases of fasciolosis and to formulate suitable control strategies.

## Data Availability

All data generated or analyzed during this study are included in the manuscript.

## Supplementary material

*Supplementary Table 1*: Specific primers targeting *ITS1* and *pepck* of liver fluke. *Supplementary Table 2*: Assessment guidelines for viability scoring.

The supplementary material of this article is available at https://www.parasite-journal.org/10.1051/parasite/2026004/olm.
