# Supplementary material for: Fasciolosis, a foodborne zoonotic trematode infection in cattle in Bangladesh: multifaceted validation of parthenogenecity and anthelmintic efficacy
Source: Parasite. 2026 Feb 11;33:7. doi: 10.1051/parasite/2026004 (PMC12892867; doi:10.1051/parasite/2026004)
Supplement: Supplementary file 1 — Supplementary Table 1: Specific primers targeting ITS1 and pepck of liver fluke. [file parasite-33-7-s1.pdf]

Supplementary Table 1: Specific primers targeting *ITS1* and *pepck* of liver fluke

	Gene	Sequences (5′-3′)	Size(bp)	Ref.
ITS1-f	*ITS1*	TTGCGCTGATTACGTCCCTG	680	[10]
ITS1-r		TTGGCTGCGCTCTTCATCGAC		
Fh-Pepck-F	*pepck*	GATTGCACCGTTAGGTTAGC	241 bp for *F. hepatica,* 510 bp for *F. gigantica*	[38]
Fg-Pepck-F		AAAGTTTCTATCCC GAACGAAG		
Fcmn-Pepck-R		CGAAAATTATGGCATCAATGGG		
