# Supplementary material for: Fasciolosis, a foodborne zoonotic trematode infection in cattle in Bangladesh: multifaceted validation of parthenogenecity and anthelmintic efficacy
Source: Parasite. 2026 Feb 11;33:7. doi: 10.1051/parasite/2026004 (PMC12892867; doi:10.1051/parasite/2026004)
Supplement: Supplementary file 2 — Supplementary Table 2: Assessment guidelines for viability scoring. [file parasite-33-7-s2.pdf]

Supplementary Table 2: Assessment guidelines for viability scoring

Sl. no.	Score	Grading standards
1	00	Dead parasites, no movement, heavy granulation, rough outer tegument and blebs
2	01	Very reduced motility, rough outer tegument with some blebs
3	02	Reduced motility, slight granularity, intact tegument with slight deformations and few blebs.
4	03	Regular contractions, a smooth outer surface, no granulation with clear view of internal structures but very few blebs or infrequent blebs.
5	04	Regular smooth contractions , no blebs and a smooth outer surface, no granulation, flow of internal contents with clear view of internal structures
